# PP/TiO_2_ Melt-Blown Membranes for Oil/Water Separation and Photocatalysis: Manufacturing Techniques and Property Evaluations

**DOI:** 10.3390/polym11050775

**Published:** 2019-05-01

**Authors:** Fei Sun, Ting-Ting Li, Haitao Ren, Qian Jiang, Hao-Kai Peng, Qi Lin, Ching-Wen Lou, Jia-Horng Lin

**Affiliations:** 1 Innovation Platform of Intelligent and Energy-Saving Textiles, School of Textile Science and Engineering, Tianjin Polytechnic University, Tianjin 300387, China; sunfei_92@163.com (F.S.); renhaitaomail@163.com (H.R.); jiangqian@tjpu.edu.cn (Q.J.); skyphk@163.com (H.-K.P.); 2 Tianjin and Education Ministry Key Laboratory of Advanced Textile Composite Materials, Tianjin Polytechnic University, Tianjin 300387, China; 3 Fujian Key Laboratory of Novel Functional Fibers and Materials, Minjiang University, Fuzhou 350108, China; 4 Fujian Engineering Research Center of New Chinese Lacquer Material, Minjiang University, Fuzhou 350108, China; linqi@mju.edu.cn; 5 Department of Bioinformatics and Medical Engineering, Asia University, Taichung 41354, Taiwan; 6 Department of Medical Research, China Medical University Hospital, China Medical University, Taichung 40402, Taiwan; 7 College of Textile and Clothing, Qingdao University, Shandong 266071, China; 8 Laboratory of Fiber Application and Manufacturing, Department of Fiber and Composite Materials, Feng Chia University, Taichung 40724, Taiwan; 9 School of Chinese Medicine, China Medical University, Taichung 40402, Taiwan; 10 Department of Fashion Design, Asia University, Taichung 41354, Taiwan

**Keywords:** polypropylene, membranes, melt-blowing process, oil/water separation, photocatalysis

## Abstract

This study aims to produce polypropylene (PP)/titanium dioxide (TiO_2_) melt-blown membranes for oil/water separation and photocatalysis. PP and different contents of TiO_2_ are melt-blended to prepare master batches using a single screw extruder. The master batches are then fabricated into PP/TiO_2_ melt-blown membranes. The thermal properties of the master batches are analyzed using differential scanning calorimetry and thermogravimetric analysis, and their particle dispersion and melt-blown membrane morphology are evaluated by scanning electron microscopy. TiO_2_ loaded on melt-blown membranes is confirmed by X-ray diffraction (XRD). The oil/water separation ability of the melt-blown membranes is evaluated to examine the influence of TiO_2_ content. Results show that the thermal stability and photocatalytic effect of the membranes increase with TiO_2_ content. TiO_2_ shows a good dispersion in the PP membranes. After 3 wt.% TiO_2_ addition, crystallinity increases by 6.4%, thermal decomposition temperature increases by 25 °C compared with pure PP membranes. The resultant PP/TiO_2_ melt-blown membrane has a good morphology, and better hydrophobicity even in acetone solution or 6 h ultraviolet irradiation, and a high oil flux of about 15,000 L·m^−2^·h^−1^. Moreover, the membranes have stabilized oil/water separation efficiency after being repeatedly used. The proposed melt-blown membranes are suitable for mass production for separating oil from water in massively industrial dyeing wastewater.

## 1. Introduction

Currently, water pollution problems have given rise to strong repercussions in society, particularly for oily wastewater pollution. This considerable amount of oily wastewater usually comes from textile, petrochemical, and steel factories. Sometimes, in the marine transportation, oil spills happen by leakage, which pollutes the ocean and causes oily wastewater pollution [1,2]. Therefore, how to deal with these oily wastewater pollutions and to achieve oil/water separation have attracted the attention of researchers.

In the industry, oil/water mixtures can be purified by some mechanical devices, such as skimmers, air flotation, centrifugation, booms, and chemical coagulation. However, these purifying methods need to input energy and high pressure to operate [3,4]. Porous materials such as foam [5,6] and textiles [7,8] are often used to deal with oil leakage by adsorption. However, water and oil are both adsorbed and the separation efficiency is poor. Moreover, recovery is difficult and time consuming and even leads to secondary environmental pollution [9]. To address these issues, many scholars coated thermal- [10] or pH- [11,12] responsive materials on metallic mesh to improve oil/water separation ability and to provide different applications. Similarly, titanium meshes [13] and porous polyethylene (PE) meshes [14] that are modified with poly(3,4-ethylenedioxythiophene) -poly(styrenesulfonate) have high resistance to the harshest chemical conditions. Metallic meshes have shown promising results for the selective separation of oil/water mixtures. However, such meshes have disadvantages such as heavy weight, high material cost, and poor corrosion resistance impeding practical applications. By contrast, textiles/fabric based substrates are low cost, light weight, flexible, and corrosion resistant. Several studies focused on nanofiber membranes and their application in the separation of oil/water systems. For example, electrospun mats composed of polydimethylsiloxane (PDMS)-block-poly (4-vinylpyridine) polymers show excellent pH switchability for oil-water separation [15]. Electrospun membranes composed of a fluorine-containing polymer exhibit self-healing ability by self-cleaning, which is ascribed to the low-surface-energy of fluorine-containing polymer [16]. However, this fluorine-containing polymer has environmental damage. Preferably, TiO_2_ not only can decompose the organic pollutants on the membranes, but also demonstrate light-induced oil resistance and self-cleaning function [17,18]. As a photocatalyst, TiO_2_ decomposes organic pollutants via light-induced oxidative holes and hydroxyl radicals [19,20]. Comparatively, TiO_2_ is commonly used in the photocatalytic degradation of organic pollutants because of its superior advantages, such as low production cost, high stability, and good photocatalysis [21]. TiO_2_ also commonly used in oil/water separation. For example, depositing TiO_2_ on stainless steel meshes [22,23] or titanium meshes [18] can produce oil/water-separating membranes. A good prospect in separating oil from water effectively is achieved by adding TiO_2_ with meshes or membranes. However, these methods still have some limitations. Even under low water resistance, TiO_2_ and meshes present a low adhesion between them. Therefore, ideal photocatalyst-immobilized substrate materials should be selected for stable anchoring to prevent catalyst leaching, maintain reactive oxygen stability, and increase selective affinity toward target contaminants [24].

To overcome the aforementioned shortcomings of metal mesh and electrospinning, previous studies employed an effective melt-blown method to form microfiber membranes and improve TiO_2_ adhesion [25,26,27]. As a commonly available low-cost melt-blown substrate, polypropylene (PP) exhibits efficient air filtration performance and water purification [28,29,30,31]. Liu et al. [28] found that melt-blown PP membrane significantly decreased the amount of chemical oxygen demand (COD), suspended solids (SS), and NH_3_-N in the reclaimed water. Moreover, some particles, such as nanoclay [29], silicon dioxide [30], and tourmaline particles [31] can be added in the melt-blowing process in order to create additional new function in a greater diversity application. In addition, melt-blending process is an efficient method to produce massive functional matrix, featuring high production efficiency, less pollution, and ease of processing. Moreover, some particles, such as nanoclay [29], silicon dioxide [30], and tourmaline particles [31], can be added in the melt-blowing process to create new functions for different applications. Melt-blending is an efficient method to produce massive functional matrices featuring high production efficiency, less pollution, and ease of processing. Therefore, in this study, a single-screw melting blending process is first used to prepare PP/TiO_2_ blends, and then photocatalytic PP/TiO_2_ membranes are formed by melt-blowing. The TiO_2_ dispersion, thermal stability, wetting property, and photocatalytic effect and oil/water separation of PP/TiO_2_ membranes are characterized.

## 2. Experimental

### 2.1. Materials

PP (Shanghai Yiding Plastics, Shanghai, China) has a melt index (MI) of 35 g/10 min at 170 °C. TiO_2_ particle (Shandong Jiechen Chemical, Shandong, China) has an average diameter of about 100 nm. Methylene blue (Beijing Solarbio Science & Technology Co., Ltd., Beijing, China), Rhodamine B (Beijing Solarbio Science & Technology Co., Ltd.), oil red O (Beijing Solarbio Science & Technology Co., Ltd.), and organic solvents (petroleum ether, *n*-hexane, acetone) were purchased from Aladdin Chemistry Co. Ltd., Shanghai, China. All chemicals were of analytical and used as received without further purification.

### 2.2. Preparation of Melt-Blown Membranes

PP and TiO_2_ are mixed through several physical processes at mass ratios of 100/0, 100/1, 100/3, and 100/5, melted, extruded, and then pelletized for seven times using a single-screw extruder (Qingdao Keshengda Plastic Machinery, Qingdao, China) forming a PP/TiO_2_ master batch. PP/TiO_2_ master batches are prepared after seven cycles, and the screw and pelletizing speed ratio is 1:1.52. The processing parameters of the melted blends are shown in Table 1, and the prepared master batches are named PP/TiO_2_-1, PP/TiO_2_-3, and PP/TiO_2_-5 depending on the different amounts of TiO_2_. Some master batches are then poured into a twin-screw mixer at 180 °C for 3 min at a rotary speed of 130 rpm. The obtained blends are then hot-pressed at 160 °C for 2 min under a pressure of 20 MPa to obtain PP/TiO_2_ hot-pressed films. The hot-pressed films are named F-PP/TiO_2_-0, F-PP/TiO_2_-1, F-PP/TiO_2_-3, and F-PP/TiO_2_-5 depending on the different amounts of TiO_2_.

**Figure 1:**
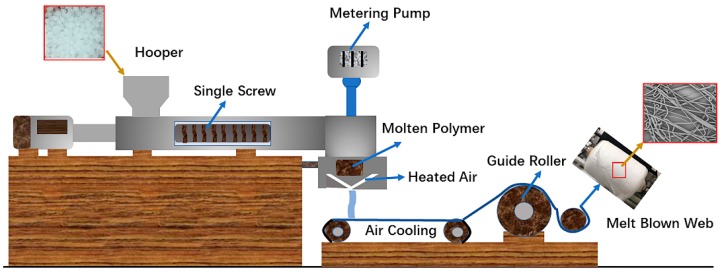
The melt-blowing process of polypropylene/titanium dioxide (PP/TiO_2_) melt-blown membranes.

The master batches are dried at 80 °C for 10 h and then poured into a melt-blowing machine (Tianjin Shengruiyuan Machinery Technology, Tianjin, China) with a die temperature of 180–230 °C (Table 1), a hot air temperature of 180 °C, and a pressure of 0.04 MPa. The melt-blowing process is shown in Figure 1. The metering pump frequency is 5 Hz, and the distance between the die and the collector is 20 cm. The membranes are denoted as M-PP/TiO_2_-0, M-PP/TiO_2_-1, M-PP/TiO_2_-3, and M-PP/TiO_2_-5 depending on the different amounts of TiO_2_.

### 2.3. Measurements and Characterizations

The thermal properties of the PP/TiO_2_ master batches are analyzed using differential scanning calorimetry (DSC 209F3, NETZSCH, Bavaria, Germany). All samples are heated from 25 °C to 200 °C at increments of 10 °C/min and then stored at 200 °C for 3 min to eliminate the thermal history. Afterward, the samples are cooled to 25 °C at 10 °C/min, during which the cooling curves are recorded. Samples are again heated to 200 °C at increments of 10 °C/min, during which the heating curves are recorded. All experiments are performed under nitrogen atmosphere. Thermogravimetric is conducted with a thermogravimetric analyzer (TG209F3, NETZSCH, Bavaria, Germany) with dry nitrogen gas at a flow rate of 60 mL/min. The relative mass losses of the PP/TiO_2_ master batches are recorded from 50 °C to 700 °C at a heating rate of 20 °C/min. The fractured surface morphology of the F-PP/TiO_2_ membranes and the surface morphology of the M-PP/TiO_2_ membranes are analyzed by scanning electron microscopy (SEM, TM3030, HITACHI, Tokyo, Japan) at an accelerating voltage of 3 KV. Prior to the test, the F-PP/TiO_2_ hot-press film samples are brittle fractured with liquid nitrogen, and then all samples are sputtered with gold to provide enhanced conductivity. The SEM images are analyzed using Image-Pro Plus 6.0 software. A bundle of fibers per image are used to measure the diameter of the nanofibers, and Origin is used to plot the diameter distribution and compute the standard deviations. The contact angles of the M-PP/TiO_2_ melt-blown membranes are measured using a tester JC2000DM (Powereach, Shanghai Zhongchen Digital Technology Apparatus, Shanghai, China). The contact angles at five sites of the sample are measured and recorded.

### 2.4. Oil/Water Separation and Photocatalytic Activity Analysis

The M-PP/TiO_2_ melt-blown membrane is affixed between two glass vessels. To distinguish, water is dyed with methylene blue, and organic solvents (i.e., kerosene, hexane, petroleum ether, and toluene) are dyed with oil red O. Water (30 mL) and oil (30 mL) are blended, and the mixture is gently poured into the container. Oil and water are separated under gravity, and the water/oil separation efficiency is computed using Equation (1) [32]:

η = (V_1_/V_0_) × 100, (1)

where V_0_ and V_1_ correspond to the volume of oil before and after the separation, respectively. Oil that adheres to the vessel is not included in the calculation.

The photocatalytic activities of the M-PP/TiO_2_ melt-blown membranes are evaluated using rhodamine B and kerosene. Rhodamine B has an initial concentration of 10 mg/L. Moreover, 5 mg/30 mL of TiO_2_ is used as the photocatalyst. Photocatalytic degradation of Rhodamine B is conducted using a photochemical reaction apparatus (XPA-VII photochemical reactor, Xujiang electromechanical, Nanjing, China). Ultraviolet light irradiation is sourced from a 300 W Hg lamp that resides in a double glazing jacket where circulating cool water flows. Then, a specified volume of solution is extracted at different times. The solution is first centrifuged, after which the supernatant liquor is taken and the absorbance is recorded at 554 nm using a UV-Vis spectrophotometer (Mapada, UV-1800PC, Shanghai, China). Rhodamine B has the maximum absorbance wavelength of 554 nm. 

The decomposition of kerosene is analyzed using Fourier transform infrared spectroscopy (FTIR, Nicolet iS10, Waltham, MA, USA). PP/TiO_2_ melt-blown membranes that are immersed in kerosene are exposed to a 300 W radiation from a high-pressure Hg lamp for 3 h. A specified volume of kerosene is collected and then scanned in the range of 400–4000 cm^−1^ using a FTIR instrument. Differences in the plotted spectrum are compared to evaluate the photocatalytic capabilities of the membranes.

## 3. Results and Discussion 

### 3.1. Thermal Behaviors and Thermal Stability of the PP/TiO_2_ Master Batch

**Figure 2:**
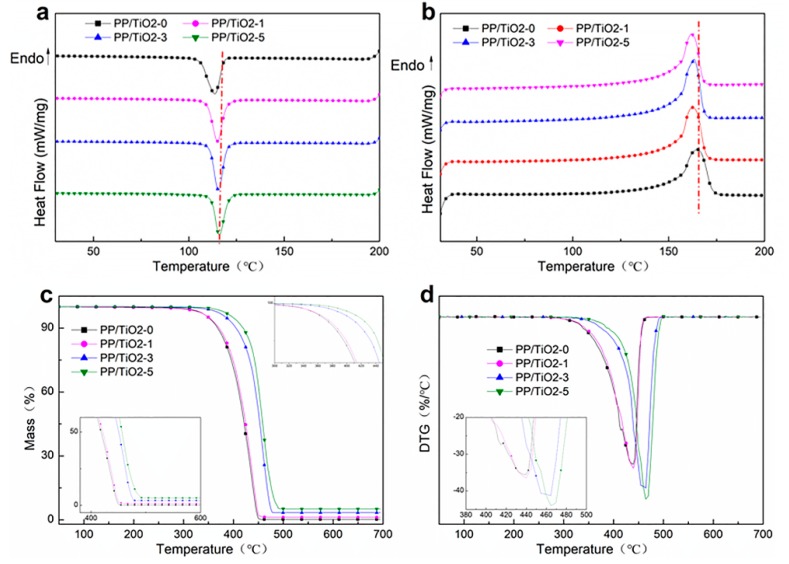
Thermal properties of PP/TiO_2_ master batch. (**a**) DSC thermograms of first cooling processes; (**b**) DSC thermograms of second heating; (**c**) TG curves of PP/TiO_2_; (**d**) DTG curves of PP/TiO_2_.

Figure 2a,b shows the DSC cooling and heating curves of the PP/TiO_2_ master batches after eliminating the thermal history. Table 2 shows the melt crystallization temperature (T_mc_), the glass transition temperature (T_g_), the melt temperature (T_m_), and the degree of crystallinity (X_c_) of the PP/TiO_2_ master batches with different TiO_2_ contents. The T_g_ of pure PP is 41 °C, which is heightened with the presence of TiO_2_. However, the content of TiO_2_ only has a marginal positive influence. During the cooling process, the master batches exhibit a cold crystallization peak. The cold crystallization peak increases with TiO_2_ content, indicating that the crystallization rate of the PP/TiO_2_ master batch. This result suggests that using TiO_2_ accelerates the crystallinity of PP. The reason is that TiO_2_ inorganic particles can serve as a heterogeneous nucleation agent. Thus, polymer crystals have changed from homogeneous nucleation to heterogeneous nucleation, facilitating the crystallization. The crystallinity of the PP/TiO_2_ master batches is proportional to the content of TiO_2_. The degree of crystallinity (X_c_) of the samples is calculated using the following equation:

X_c_ = {(ΔH_m_ − ΔH_cc_)/ΔH_0_} × 100%, (2)

where ΔH_m_ is the melting enthalpy, ΔH_cc_ is the cold crystallization enthalpy, and ΔH_0_ is the fusion enthalpy of the completely crystalline PP [33].

When the TiO_2_ content is 3 wt. %, the PP/TiO_2_-3 master batch has a crystallinity of 42.69%, which is higher than that of PP/TiO_2_-0 (36.29%). Nevertheless, greater TiO_2_ content is not beneficial to the crystallinity of PP, which suggests that increasing TiO_2_ to a certain extent decreases crystallization. As a result, the crystallinity remains the same and the heterogeneous nucleation agent is saturated.

The Thermogravimetric analysis (TGA) and differential thermogravimetry analysis (DTG) curves of the PP/TiO_2_ master batches are related to the content of TiO_2_ as shown in Figure 2c,d. The results of thermogravimetric show that the TGA curves of the PP/TiO_2_ master batches shift toward high temperature as the TiO_2_ content is increased. Table 3 shows the initial decomposition temperature (T_0.05_), a weight loss 50% temperature (T_0.5_), remains mass, and “T_max_” is the decomposition temperature corresponding to the weight loss rate obtained from the DTG curve in Figure 2d. The PP/TiO_2_ master batches decomposition temperature increases with increasing TiO_2_ content. The T_0.5_ of PP/TiO_2_-0 is 350 °C, and that of the PP/TiO_2_ master batch containing 5 wt. % TiO_2_ is 400 °C. This finding demonstrates that the presence of TiO_2_ increases the decomposition temperature and improves the thermal stability of PP/TiO_2_. This result is due to the fact that TiO_2_ can enhance the interaction between polymer molecular chains, which consequently increases the degree of crosslinking and restricts the segmental motion. The thermal decomposition of PP molecular chains is terminated, while the distribution of TiO_2_ in the PP polymer limits the transmission of heat energy [34]. In addition, the PP/TiO_2_ master batches have high remnant mass than PP/TiO_2_-0, and the remaining amount is almost equivalent to the content of TiO_2_. The greater TiO_2_ content generates the higher remnant mass.

### 3.2. Morphology of PP/TiO_2_ Hot-Press Film and Melt-Blown Membranes

**Figure 3:**
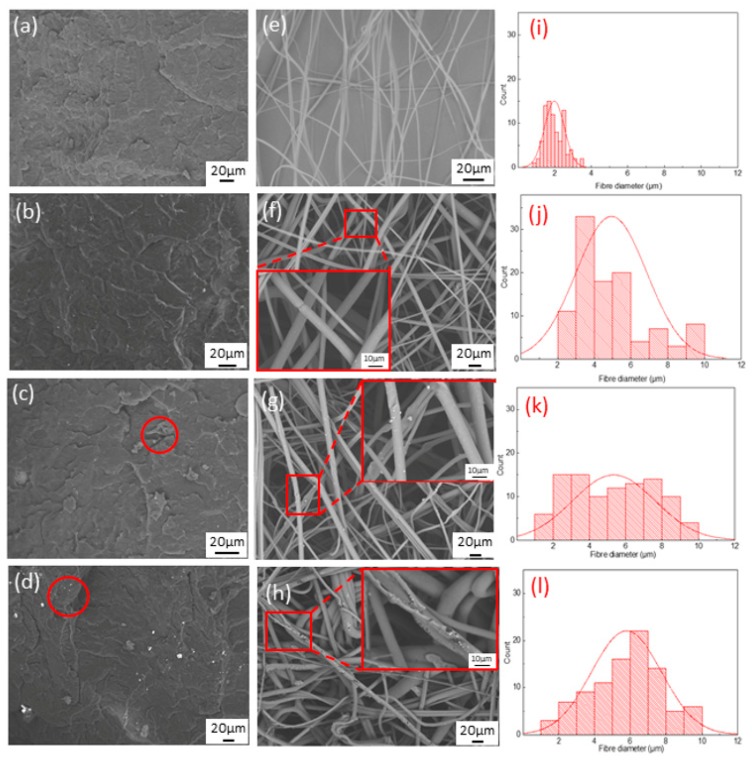
The fracture surfaces of PP/TiO_2_ hot-press film (**a**) F-PP/TiO_2_-0, (**b**) F-PP/TiO2-1, (**c**) F-PP/TiO_2_-3, (**d**) F-PP/TiO_2_-5; the SEM images of PP/TiO_2_ melt-blown membranes (**e**) M-PP/TiO_2_-0, (**f**) M-PP/TiO_2_-1, (**g**) M-PP/TiO_2_-3 (**h**) M-PP/TiO_2_-5, and the fiber diameter of PP/TiO_2_ nonwovens (**i**) M-PP/TiO_2_-0, (**j**) M-PP/TiO_2_-1, (**k**) M-PP/TiO2-3, and (**l**) M-PP/TiO_2_-5.

The fractured surface of the PP/TiO_2_ hot-press film and the surface morphology of the PP/TiO_2_ melt-blown membranes are shown in Figure 3a–d. The fractured surface of PP/TiO_2_-0 is continual and homogeneous. By contrast, the PP/TiO_2_ hot-press film has a rugged fractured surface where the white particles are TiO_2_. During melt-blending, some agglomerated particles can be separated by a strong shear force effectively [35]. However, increasing the content of TiO_2_ leads to agglomeration in the polymer matrix, and the agglomeration becomes more apparent with increasing TiO_2_ content, suggesting that the adhesion between PP and TiO_2_ is low. Some of the TiO_2_ particles fall apart from the membranes because of the brittle fracture caused by liquid nitrogen, leaving some empty holes. This phenomenon is more exaggerated as the TiO_2_ content is increased 

Figure 3e–h shows the fiber morphology of the PP/TiO_2_ melt-blown membranes with the corresponding fiber diameter distribution (Figure 3i–l). Table 4 shows the average diameter and standard deviation of the membranes. M-PP/TiO_2_-0 is composed of fibers with an average diameter of about 2 µm and has a smooth surface. With the addition of TiO_2_, M-PP/TiO_2_ is composed of fibers with different diameters, and some fibers are coalesced obviously, which is ascribed to the presence of TiO_2_. During melt-blowing, TiO_2_ of a small particle size is enwrapped in the fibers, whereas some agglomerated TiO_2_ particles protrude the fibers and are responsible for the rough fiber surface.

### 3.3. XRD and FTIR Analysis of PP/TiO_2_ Melt-Blown Membrances

**Figure 4:**
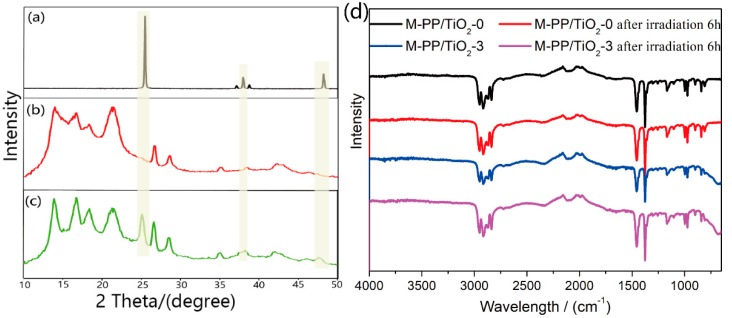
X-ray diffraction (XRD) patterns of (**a**) TiO_2_; (**b**) M-PP/TiO_2_-0; (**c**) M-PP/TiO_2_-3; and (**d**) Fourier transform infrared spectroscopy (FTIR) spectra of M-PP/TiO_2_ membranes.

The X-ray diffraction (XRD) patterns of TiO_2_, M-PP/TiO_2_-0, and M-PP/TiO_2_-3 are shown in Figure 4a–c. The XRD pattern of M-PP/TiO_2_-0 predominantly consists of α-phase with (110), (040), and (130) characteristic peaks at 2θ = 14°, 17°, and 18.6°, respectively (Figure 4b,c). TiO_2_ has (101), (004), and (200) characteristic peaks corresponding to 25.37°, 37.88°, and 48.12° (Figure 4a). All of these characteristic peaks can be observed from M-PP/TiO_2_-3 in Figure 4c. This result indicates that TiO_2_ is successfully loaded on the melt-blown membrane. The surface composition of the membranes is further analyzed by FTIR, as shown in Figure 4d. For the M-PP/TiO_2_ membranes, the peaks at 2950 and 2917 cm^−1^ are respectively assigned to the –CH_3_ and –CH_2_– asymmetric stretching vibrations, respectively; those at 2873 and 2840 cm^−1^ correspond to the –CH_3_ and –CH_2_– symmetric stretching vibrations, respectively; and those at 1459 and 1377 cm^−1^ correspond to –CH_2_– bending vibration and –CH_3_ symmetric deformation vibration, respectively. No additional XRD and FTIR characteristic peaks are found, which shows that the incorporation of TiO_2_ cannot change the crystal structure and chemical groups of the membrane compared with M-PP/TiO_2_-0. 

### 3.4. Oil/Water Separation of PP/TiO_2_ Melt-Blown Membranes

**Figure 5:**
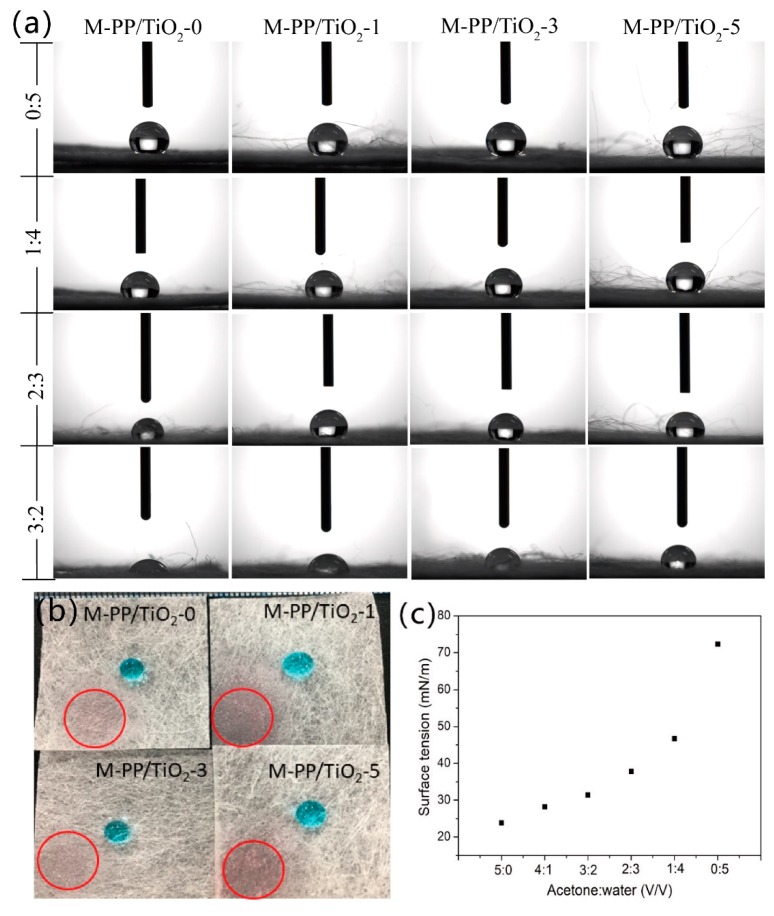
(**a**) Contact angle of M-PP/TiO_2_ (m:n represent acetone/water (*v/v*)); (**b**) the oil and water drip on the melt-blown membrane (the red circle is kerosene, blue drop is water), and (**c**) the surface tension of different acetone solution.

A high selective wettability for oil and water is another crucial factor for oil/water separation materials [36]. The wettability of M-PP/TiO_2_ is determined by the water contact angle measurements. Figure 5a shows the contact angle observation of the membranes, and the accurate contact angle is shown in Table 5. M-PP/TiO_2_-0 has a contact angle of 140°, representing good hydrophobicity. Although high TiO_2_ contents decrease the water contact angle, it remains beyond 130°. Therefore, the membranes possess hydrophobicity, and the presence of TiO_2_ does not impact their wettability. Moreover, the oil (i.e., kerosene) and organic solvent (i.e., petroleum ether, *n*-hexane, and acetone) contact angle of the membranes is 0° (Figure 5b), suggesting super lipophilicity. The contact angle of the acetone drops on the filtration membrane gradually increases with increasing TiO_2_ content. When the ratio of acetone and water is 3:2, the contact angle of acetone solution in M-PP/TiO_2_-0 is 20°, whereas that in M-PP/TiO_2_-3 is 98°. The surface tension of the acetone-simulated organic pollutant solution is measured using a liquid surface tension tester (BZY-3B, Shanghai Hengping Instrument and Meter Factory) to confirm the interaction of TiO_2_ in the membrane and operating environment at organic pollutants. Figure 5c shows the surface tension of different acetone solutions. The surface tension of the solution decreases gradually with increasing acetone concentration, whereas the surface tension of the membranes decreases gradually with increasing TiO_2_ content to meet the different surface tensions of liquid filtration.

According to Young equation, the wetting angle of a droplet is related to the surface tension of the solid–gas, liquid–gas, and liquid–solid interfaces. For the same droplet, the liquid surface tension is constant. The greater the contact angle, the greater the difference between the surface tension of the solid–gas and solid–liquid interfaces. As a result, the introduction of TiO_2_ changes the surface tension of the membranes.

(3) $$\gamma_{sg}-\gamma_{sl}=\gamma_{lg}cos\theta ,$$

where $\gamma_{sg}$ is the surface tension of the solid–gas interface, $\gamma_{sl}$ is the surface tension of the solid–liquid interface, $\gamma_{lg}$ is the surface tension of the liquid–gas interface, and θ is the contact angle.

**Figure 6:**
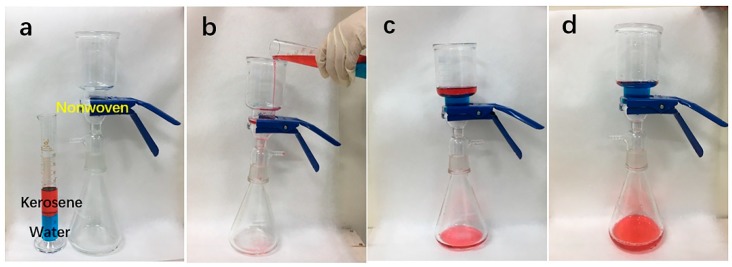
The oil-water separation process of M-PP/TiO_2_.

M-PP/TiO_2_ exhibits both hydrophobicity and super lipophilicity and thus can be applied to oil/water separation. A simple oil/water separation equipment is assembled, and its separation process is shown in Figure 6. The membrane is affixed between two glass containers, after which the water/kerosene mixture is poured into the upper container slowly. With the help of gravity, kerosene can permeate through the membrane and reach the lower glass container, during which water is intercepted and stays over the melt-blown membrane. No other external forces are applied. As a result, oil is separated from water effectively, which proves that the separation process can be easily conducted with a low energy cost.

**Figure 7:**
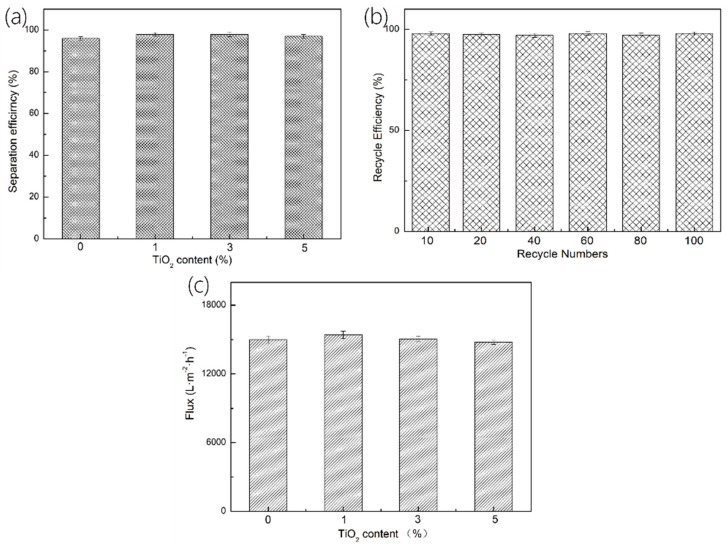
(**a**) Oil/water separation efficiency (ηs) of melt-blown membranes as related to TiO_2_ content, (**b**) oil/water separation efficiency of M-PP/TiO_2_-3 as related to recycle numbers, and (**c**) gravity-driven kerosene flux of melt-blown membranes as related to TiO_2_ content.

Afterward, the oil/water separation performance of M-PP/TiO_2_ is computed to obtain the oil–water separation efficiency (η_s_). Figure 7a shows that the separation efficiency of the membranes is influenced by TiO_2_ content. The separation efficiency is between 95–98%, suggesting effective oil/water separation. Noticeably, the membranes retain their oil/water separation capability even after 100 repeated tests. The test result proves that the proposed membranes have a high stability (Figure 7b). Importantly, the separation efficiency is not dependent on the TiO_2_ content when the test is conducted under gravity. The oil flux (F) can be yielded using the following Equation (3):

(4) $$F=\frac{V}{St},$$

where *V* is the volume of kerosene (i.e., *V* = 50 mL), *S* is the effective geometric area (1256 mm^2^), and *t* is the time required. 

The oil flux of the PP/TiO_2_ membranes is 14,789–15,410 L·m^−2^·h^−1^ as shown in Figure 7c, which is higher than those of the stainless filtering net-based membranes and electrospinning membranes [37].

### 3.5. Photocatalysis of PP/TiO_2_ Melt-Blown Membranes

**Figure 8:**
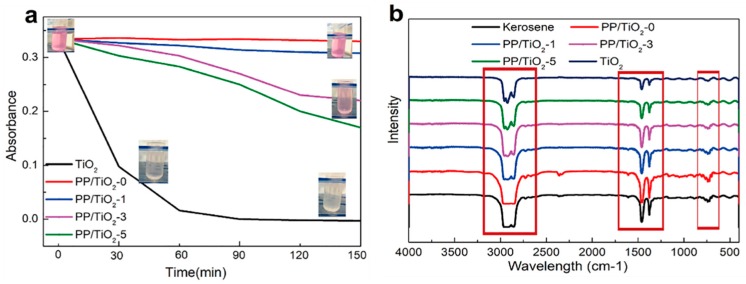
(**a**) The absorbance of rhodamine B along with time and (**b**) FTIR spectrum of kerosene photocatalysis PP/TiO_2_ melt-blown membranes.

Figure 8 shows the photocatalysis of the F-PP/TiO_2_ melt-blown membranes. Rhodamine B solution almost does not decompose under UV light irradiation [38]. Therefore, rhodamine B is selected as a model organic pollutant to study the photocatalytic performance of the F-PP/TiO_2_ membranes. Figure 8a shows the photocatalysis of the melt-blown membrane-decomposed rhodamine B solution. The melt-blown membranes composed of high TiO_2_ amounts demonstrate greater photocatalysis, which is lower than that of pure TiO_2_. This phenomenon can be attributed to two reasons. For one thing, the melt-blown membranes are hydrophobic, and the polluted sample and photocatalyst may not have sufficient contact during the photocatalysis. For another, some TiO_2_ particles are enwrapped in the PP melt-blown membranes or fibers, which deprives TiO_2_ of its possibility to directly contact with light sources. Therefore, the membranes demonstrate a low photocatalysis. 

Kerosene is used as the contaminant to testify the major factor affecting the photocatalysis of the membranes. The membranes possess a good lipophilic property and thus have good wettability. Figure 8b shows the spectrum of kerosene before/after photocatalysis. Kerosene demonstrates C–H stretching at 3000–2850 cm^−1^ and C–H vibration at 1465–1340 cm^−1^, which indicates strong characteristic peak. When the TiO_2_ content is increased, the intensity of the characteristic peak gradually declines. The photocatalysis of the PP/TiO_2_ melt-blown membranes is dependent on the content of TiO_2_ distributed over the fibers. When the TiO_2_ content is 3% or 5%, the photocatalytic effect is similar, which proves that the TiO_2_ content in the membranes is similar. Combined with the SEM image (Figure 3), when the TiO_2_ content is 3%, the membranes show higher photocatalytic effect and better morphology.

## 4. Conclusions

In this study, TiO_2_ and PP are melt-blended to form PP/TiO_2_ master batches using a single-screw extruder, after which the master batches are prepared into PP/TiO_2_ melt-blown membranes. The thermal properties of PP/TiO_2_ master batches and the melt-blown membrane morphology are characterized. DSC shows that the presence of TiO_2_ accelerates crystallization rate, providing the master batch with high crystallinity. Similarly, TGA results show that the presence of TiO_2_ enhances the thermostability of PP, and the thermal decomposition temperature is proportional to the content of TiO_2_ and reaches the required temperature of 180–230 °C. SEM images show that TiO_2_ is evenly dispersed in the PP matrices due to multiple melt-blending processes. In addition, TiO_2_ provides the fibers with a large diameter and a rough surface, which makes the fiber web fluffy. TiO_2_ also contributes photocatalytic activity to the membranes and a great amount of kerosene flux, leading to high water/oil separation efficiency. Moreover, the membranes can be used repeatedly and maintain stabilized filtering efficacy. The membranes remain stable after 6 h of ultraviolet irradiation, indicating that TiO_2_ does not degrade PP. The proposed melt-blown membranes are suitable for mass production for separating oil from water in massively industrial dyeing wastewater.

## Figures and Tables

**Table 1:** The Processing parameters of melt-blending.

Parameter	Screw 1 Temperature (°C)	Screw 2 Temperature (°C)	Screw 3 Temperature (°C)	Nozzle Temperature (°C)	Screw Speed (r/min)	Pelletized Speed (r/min)
	180	200	190	170	21	32

**Table 2:** Differential scanning calorimetry (DSC) results of melt-blown membranes.

Sample	T_mc_ (°C)	T_g_ (°C)	T_m_ (°C)	X_c_ (%)
PP/TiO_2_-0	113.60	41.50	164.90	36.29
PP/TiO_2_-1	115.00	42.40	162.50	37.68
PP/TiO_2_-3	115.80	42.20	163.30	42.69
PP/TiO_2_-5	116.00	42.10	161.90	40.81

**Table 3:** TGA and DTG results of PP/TiO_2_ melt-blown membranes.

Sample Type	T_0.05_ (°C)	T_0.5_ (°C)	T_max_ (°C)	Remnant Mass (%)
PP/TiO_2_-0	349.80	418.13	438.60	0.02
PP/TiO_2_-1	350.70	421.30	440.40	0.84
PP/TiO_2_-3	385.50	450.40	462.60	2.95
PP/TiO_2_-5	399.30	456.60	466.60	5.11

**Table 4:** Diameter distribution of melt-blown membranes as related to the content of TiO_2_.

Sample	N Total	Mean	Standard Deviation	Sum (μm)	Minimum (μm)	Median (μm)	Maximum (μm)
M-PP/TiO_2_-0	88	1.99	0.54	175.37	0.94	1.86	3.56
M-PP/TiO_2_-1	104	4.93	1.93	512.80	2.46	4.43	9.72
M-PP/TiO_2_-3	99	5.34	2.26	529.08	1.74	5.22	9.91
M-PP/TiO_2_-5	93	5.78	1.95	537.76	1.74	6.09	9.84

**Table 5:** The contact angle of acetone solution at PP/TiO_2_ melt-blown membranes.

Acetone:Water (*v*/*v*)	M-PP/TiO_2_-0	M-PP/TiO_2_-1	M-PP/TiO_2_-3	M-PP/TiO_2_-5
0:5	140	138	134	132
1:4	116	130	132	132
2:3	109	116	120	124
3:2	20	59	76	98
